# Language Impairments in Individuals With Coffin-Siris Syndrome

**DOI:** 10.3389/fnins.2021.802583

**Published:** 2022-01-20

**Authors:** Ashley Vasko, Samantha A. Schrier Vergano

**Affiliations:** ^1^Clinical Research Unit, Children’s Hospital of the King’s Daughters, Norfolk, VA, United States; ^2^Division of Medical Genetics and Metabolism, Children’s Hospital of the King’s Daughters, Norfolk, VA, United States; ^3^Department of Pediatrics, Eastern Virginia Medical School, Norfolk, VA, United States

**Keywords:** Coffin-Siris syndrome (CSS), language, BAF complex, intellectual and developmental disabilities, speech

## Abstract

Coffin-Siris syndrome (CSS, MIM 135900) is a now well-described, multiple congenital anomaly/intellectual disability syndrome classically characterized by fifth digit/nail hypoplasia, coarse facial features, and a range of organ-system related anomalies. Since its initial description in 1970, and the discovery of associated genes in 2011, CSS now encompasses a wide range of phenotypes and abilities caused by pathogenic variants in the BAF complex (often referred to as “BAFopathy”). It appears that the BAF complex leads to speech and language impairments in this population, and subsequently we have reviewed individuals in the CSS/BAF registry to understand the prevalence and degree of this particular learning difference. We have examined the frequency of delayed language acquisition, augmented communication device use, and speech intervention therapies. To aid in language progression, childhood speech interventions are necessary in children with a diagnosis of CSS. While the majority of children with pathogenic variants in the BAF complex have language-related struggles, the exact mechanism is not yet fully understood. At the time of writing, there are 284 individuals in the CSS/BAF registry with known variants in the following genes; *ARID1B* (*n* = 174), S*MARCA4* (*n* = 41), *ARID1A* (*n* = 20), *SMARCB1* (*n* = 20), *ARID2* (*n* = 14), *SOX11* (*n* = 10), and *SMARCE1* (*n* = 5). While speech delays in individuals with CSS are expected, a full analysis of these delays has yet to be detailed. In the CSS/BAF registry, we identified 183 (64%) individuals with language-related challenges and 90 (32%) individuals that are non-verbal.

## Introduction

Genes associated with Coffin-Siris syndrome (CSS) are components of the BAF chromatin remodeling complex. Proteins involved in the BAF complex are responsible for regulating chromatin structure and transcription. In the current literature, the BAF complex is felt to be responsible for gene expression and differentiation, particularly in the brain (Alfert et al., 2019). Subsequently we have theorized that a pathogenic change in this complex may impact language development, causing varying magnitudes of speech delays. Genes associated with the BAF complex and CSS include *ARID1A*, *ARID1B*, *ARID2*, *DPF2*, *SMARCA4*, *SMARCB1*, *SMARCC2*, *SMARCE1*, *SOX11*, and *SOX4* (Hempel et al., 2016; Jung et al., 2017; Vasileiou et al., 2018; Vergano et al., 2018). Pathogenic variants in the BAF complex are known to cause CSS, Nicolaides-Baraitser syndrome (NCBRS, MIM 601358), Borjeson-Forssman-Lehmann syndrome (BFLS, OMIM 301900), and other CSS-like conditions (*BICRA* and *SMARCD1*) (Wieczorek et al., 2013; Kosho and Okamoto, 2014). Although different, these conditions are all clinically similar to one another. Each of these conditions present themselves as varying magnitudes of developmental delays and intellectual disabilities. Likewise, CSS is associated with a variety of clinical neurologic presentations including intellectual disability (ID), developmental delays, autism spectrum disorder (ASD), and attention deficit hyperactivity disorder (ADHD), in addition to organ-system abnormalities (Santen and Clayton-Smith, 2014; Tsurusaki et al., 2014; Bögershausen and Wollnik, 2018).

While the association between CSS and delayed language acquisition have been reported, the magnitude and details of these delays has not been assessed. We used parent-completed questionnaire responses to determine the frequency and severity of language delays in 246 individuals in the CSS/BAF registry. Although we had access to 246 parent-completed questionnaires, we only had full medical records for 97 of them. In our patient population, we were able to substantiate the severity of these language delays. This report identifies the specific challenges that CSS children face regarding language acquisition and expression, in an effort to provide families and clinicians with a better understanding of how to navigate these challenges.

## Materials and Methods

The Coffin-Siris/BAF-related disorders registry (IRB #15-03-EX-0058), created in 2015, includes individuals with a molecular confirmation of CSS or BAF-related disorders. Individuals have varying demographics and genotypes encompassing the BAF complex. Questionnaires were piloted to gather as much valuable information on each individual as possible and were designed for parental completion. Data obtained through parent-completed questionnaires were then validated by cross-referencing the results to their medical records. The CSS/BAF-related disorders registry incorporates retrospective and prospective medical data on individuals with a molecular confirmation of CSS and aims to look at the growth, development, and medical challenges of these individuals. Individuals were included in this particular cohort if they had a molecular confirmation of CSS, a history of available developmental evaluations, and parent-completed questionnaires. In order to be included in this analysis, their developmental evaluations had to assess speech milestone achievement, and therapy modalities.

To be included in the CSS/BAF registry and the questionnaires distributed, each family was asked to sign an informed consent form prior to study participation. Study data were collected, de-identified, and managed using REDCap electronic data capture tools hosted at Children’s Hospital of The King’s Daughters (Harris et al., 2009). REDCap (Research Electronic Data Capture) is a secure, web-based application designed to support data capture for research studies, providing (1) an intuitive interface for validated data entry; (2) audit trails for tracking data manipulation and export procedures; (3) automated export procedures for seamless data downloads to common statistical packages; and (4) procedures for importing data from external sources. All study procedures and data collection were in compliance with local laws and the EVMS Investigational Review Board to protect the health and welfare of human research participants.

To determine the frequency of language delays, we evaluated 246 parent-reported questionnaires to assess the age (in months) that each individual said their first word. The Denver Scale was utilized by the study team to determine the severity of language delays, dependent on the results of the parent-completed questionnaires. According to the Denver Scale, infants typically repeat words by 12 months and say single words by 18 months. We used this criterion to separate our cohort into three groups, “normal speech development,” “moderately delayed speech development,” and “severely delayed speech development.” We defined “normal speech development” as a child who said their first word by 18 months, “mildly delayed” as a child who said their first word between 19 and 21 months, “moderately delayed speech development” as a child who said their first word between 22 and 25 months, and “severely delayed speech development” as children who said their first word after 25 months. Children that never developed speech or have a non-verbal diagnosis were included in the “severely delayed speech development” cohort. We also evaluated medical records to determine whether expressive “output” speech or receptive “input” speech was most limited.

## Results

Pathogenic variants in the BAF pathway have been associated with a variety of neurological delays. Speech development and articulation remains one of the most prevalent complaints amongst the patient population. Pathogenic variants in *ARID*-related genes account for 73% of the CSS/BAF registry population, while *SMARC*-related variants account for 26% of the CSS/BAF-related disorders registry population. Due to the significant differences in the proportion of individuals with *ARID* and *SMARC*-related variants in the BAF complex, we were unable to accurately analyze the two. To mitigate this limitation, we evaluated every individual with a molecular confirmation of CSS, regardless of exact genotype or location. We determined that 63 (26%) individuals have “normal” speech development, 2 (<1%) have mildly delayed speech development, 32 (13%) have “moderately delayed” speech development, and 149 (60%) have “severely delayed” speech. A total of 30 (12%) individuals have not a met language milestone at the time of analysis and remain non-verbal. The median age of non-verbal participants in the CSS/BAF registry is 10 years.

### Classification of Speech Complications

We were only able to assess full developmental records in 97 individuals included in the CSS/BAF registry. While a majority of individuals with CSS experience challenges in both receptive and expressive speech, 63 (65%) individuals in our cohort have more limited expressive speech versus receptive. Only one (1%) individual has more limited receptive speech and the other 33 (34%) have unspecified speech-related complications. The records supporting limited expressive speech indicate that these individuals are able to follow directions and simple commands but have difficulty communicating with their peers.

### Non-verbal Communication Methods

Verbal intelligence and language-based reasoning are standard measures of intelligence (Swillen et al., 1995). Individuals lacking verbal communication encounter unique challenges in social and academic settings. As an alternative to verbal communication, individuals may utilize augmentative and alternative communication (AAC) devices, sign language, and hand gestures (i.e., pointing) to communicate effectively. Babbling is also used as an alternative to verbal communication. In individuals with CSS, babbling is not simply utilized as a precursor to normal speech development. In our cohort, babbling is the primary means of communication in individuals as old as 14 years. We examined individual medical records to assess the number of patients that utilize an alternative form of communication. We were able to assess the AAC methods in 97 individuals included in the CSS/BAF registry. We found that babbling, as an alternative to verbal communication, is utilized in 18 (19%) individuals in our sample cohort, 21 (22%) individuals utilize an AAC device as an alternative to verbal communication, nine (9%) individuals utilize sign language, and six (6%) individuals utilize hand gestures.

### Neurological Associations

We were interested in determining whether the language impairments were also associated with a neurological diagnosis. We reviewed the MRI and neuropsychological evaluation records in 97 individuals to determine the frequency of brain malformations in individuals with CSS. We found that 17 (18%) individuals have confirmed agenesis of the corpus callosum, seven (7%) have colpocephaly, three (3%) have a mega cisterna magna, and three (3%) have ventriculomegaly. We did not have sufficient data to correlate these MRI findings with the degree of speech delays. We further evaluated these records from a neuropsychological perspective to evaluate the frequency of a neuropsychological diagnosis. Along with a molecular diagnosis of CSS, we discovered that 44 (45%) individuals have a clinical diagnosis of ASD. Neurological conditions, accompanying a CSS diagnosis, could contribute to the severity of language delays. More detailed data analysis would be required to make that determination.

## Discussion

Current literature on CSS identifies language impairments as a typical characteristic associated with pathogenic variants in the BAF complex. The purpose of this study was to evaluate the speech delays and impairment in individuals with CSS, while also developing a plan to assist caregivers in mitigating these challenges. Our study revealed that 86% of individuals in the CSS/BAF registry experienced some magnitude of language delays, which amplified the necessity for standard care efforts to improve these statistics.

Early evaluation of speech and initiation of speech therapy should be utilized as standard of-care practice in individuals with CSS. In our CSS population, 83% of individuals regularly attend speech therapy. Early speech intervention programs are effective in enhancing a child’s speech progression. To optimize speech progression, speech intervention therapies should be introduced at a young age in individuals with a CSS diagnosis. A diagnosis of CSS presents with additional clinical challenges, aside from speech. Additional therapies may be necessary to aid in progression of these delays.

A correlation between CSS and neurological impairments have been observed in our study population. These impairments can cause cognitive deficits that negatively influence speech progression and intelligibility. Seeking a differential diagnosis for cognitive deficits is recommended for individuals with CSS. Proper diagnosis and treatment plans can effectively improve a child’s development.

The inability to communicate effectively can lead to frustration for both the affected individual and their family. The frustration of communicating non-verbally can lead to aggressive and unwanted behaviors. In our cohort, a majority of families admitted to observing aggressive tendencies, in their family member with CSS, provoked by a language-related defeat. AAC devices were developed to minimize the frustration caused by language barriers. These devices can motivate a child to communicate more effectively and build confidence. For individuals with CSS, struggling with language progression, AAC devices are effective and available.

## Data Availability Statement

The original contributions presented in the study are included in the article/supplementary material, further inquiries can be directed to the corresponding author.

## Ethics Statement

The studies involving human participants were reviewed and approved by the Eastern Virginia Medical School Institutional Review Board (IRB #15-03-EX-0058). Written informed consent to participate in this study was provided by the participants’ legal guardian/next of kin.

## Author Contributions

SS and AV contributed to the conception, design, and writing of the first draft, and reviewed and edited the final draft. AV performed analyses of the data. Both authors agreed to be accountable for the content of the work herein.

## Conflict of Interest

SS is a medical advisor for the Coffin-Siris Syndrome Foundation but has not received any funding for this work. The remaining author declares that the research was conducted in the absence of any commercial or financial relationships that could be construed as a potential conflict of interest.

## Publisher’s Note

All claims expressed in this article are solely those of the authors and do not necessarily represent those of their affiliated organizations, or those of the publisher, the editors and the reviewers. Any product that may be evaluated in this article, or claim that may be made by its manufacturer, is not guaranteed or endorsed by the publisher.
